# Imaging in focus: An introduction to denoising bioimages in the era of deep learning

**DOI:** 10.1016/j.biocel.2021.106077

**Published:** 2021-11

**Authors:** Romain F. Laine, Guillaume Jacquemet, Alexander Krull

**Affiliations:** a MRC Laboratory for Molecular Cell Biology, University College London, London WC1E 6BT, UK; b The Francis Crick Institute, London NW1 1AT, UK; c Turku Bioscience Centre, University of Turku and Åbo Akademi University, 20520 Turku, Finland; d Åbo Akademi University, Faculty of Science and Engineering, Biosciences, 20520 Turku, Finland; e Turku Bioimaging, University of Turku and Åbo Akademi University, 20520 Turku, Finland; f School of Computer Science, University of Birmingham, Edgbaston, Birmingham, B15 2TT, UK

**Keywords:** Deep learning, Denoising, Live-cell imaging, Noise, Microscopy

## Abstract

Fluorescence microscopy enables the direct observation of previously hidden dynamic processes of life, allowing profound insights into mechanisms of health and disease. However, imaging of live samples is fundamentally limited by the toxicity of the illuminating light and images are often acquired using low light conditions. As a consequence, images can become very noisy which severely complicates their interpretation. In recent years, deep learning (DL) has emerged as a very successful approach to remove this noise while retaining the useful signal. Unlike classical algorithms which use well-defined mathematical functions to remove noise, DL methods learn to denoise from example data, providing a powerful content-aware approach. In this review, we first describe the different types of noise that typically corrupt fluorescence microscopy images and introduce the denoising task. We then present the main DL-based denoising methods and their relative advantages and disadvantages. We aim to provide insights into how DL-based denoising methods operate and help users choose the most appropriate tools for their applications.

## Highlights

• For live imaging, low exposure microscopy acquisitions are preferred to avoid photobleaching and sample toxicity.
• A high level of noise makes further image analysis and understanding of the microscopy images difficult and less robust.
• Supervised DL methods provide robust performance but require the curation of paired low and high-quality images.
• Self-supervised DL methods provide an easy way to denoise images as they do not require the user to provide a paired dataset.
• Recent DL methods combine denoising with other computational tasks such as segmentation or deconvolution.

## 1 Introduction

**Fig. 1:**
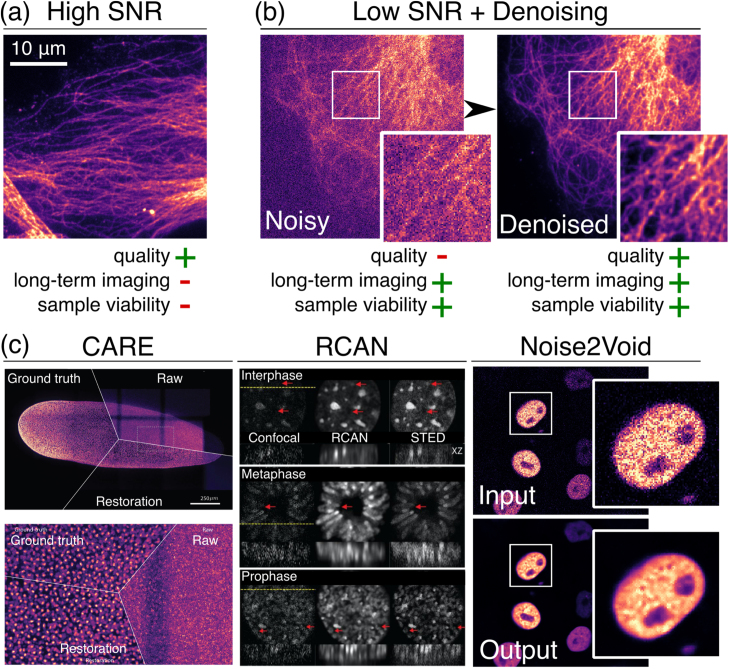
Denoising is a critical image processing tool for live fluorescence imaging. (a) The acquisition of high SNR images provides high-quality images but typically limits long-term imaging and sample viability. (b) Acquiring noisy data by using low light conditions allows for longer acquisitions due to lower phototoxicity and photodamage. Denoising can enable a more robust observation of phenomena by recovering high-quality images. (c) Examples of image denoising performed by CARE (Weigert et al., 2018), 3D RCAN (Chen et al., 2021) and Noise2Void (Krull et al., 2019) showcasing the performance of DL-based methods. SNR: Signal-to-noise ratio.

Since the beginning of fluorescence microscopy, noise has been an inevitable companion of recorded signals. In particular, live imaging often requires low illumination intensities and fast imaging, leading to the acquisition of noisy images (Fig. 1a). As a post-acquisition step, image denoising offers a powerful way to recover high-quality images and facilitate downstream analyses such as image segmentation (Fig. 1b). Many methods have been developed to reduce noise and restore the true signal. The simplest form of this is smoothing the image (i.e. Gaussian filtering), which removes noise at the expense of slightly blurring the underlying signal. Over the years, more sophisticated filtering methods have been proposed (Meiniel et al., 2018), such as, but not limited to, Non-local means (NLM) (Buades et al., 2005, Boulanger et al., 2010), block-matching 3D (BM3D) (Dabov et al., 2007) or wavelet transforms (e.g. PureDenoise (Luisier et al., 2010)). Although these advanced methods demonstrated good performance, simpler methods such as Gaussian blurring, which are commonly implemented in popular image analysis software, remain much more widely used.

Recently, *machine learning* (ML) has shown great potential for denoising (Belthangady and Royer, 2019, Moen et al., 2019), by providing high performance while avoiding effects of blurring by learning directly from the data itself. ML refers to algorithms that solve a problem from example data rather than hand-crafted mathematical procedures. An ML-based denoising system can be viewed as a highly complex mathematical function that maps a noisy image to its clean counterpart. These functions are often implemented as deep artificial neural networks (*deep learning*, DL) and can involve millions of tunable parameters. In this context, the terms *learning* or *training* correspond to the tuning of these parameters based on *training data* to improve the quality of the denoised image output. A range of methods for DL-based denoising, such as CARE (Weigert et al., 2018), 3D RCAN (Chen et al., 2021) and Noise2Void (Krull et al., 2019) (Fig. 1c), have already been demonstrated. In the context of fluorescence microscopy, these methods often outperform the best non-DL approaches when comparing common image quality metrics such as SSIM or PSNR (see Box 1 for details) (Weigert et al., 2018, Zhang, 2019).

Box 1 **Quantifying noise using image quality metrics.**

It is important to quantify the amount of noise in an image in order to compare denoising algorithm performance or simply assess the improvement observed after denoising in a specific dataset. Given a noisy image x and its corresponding clean counterpart s a standard approach is to compute the *mean squared error* (MSE) defined as

$$\mathrm{MSE}(x,s)=\frac{1}{n}\sum_{i=1}^{n}{\left(x_{i}-s_{i}\right)}^{2}$$

where the squared difference is averaged over all pixels i of the image, containing a total of n pixels.

**Fig. 2:**
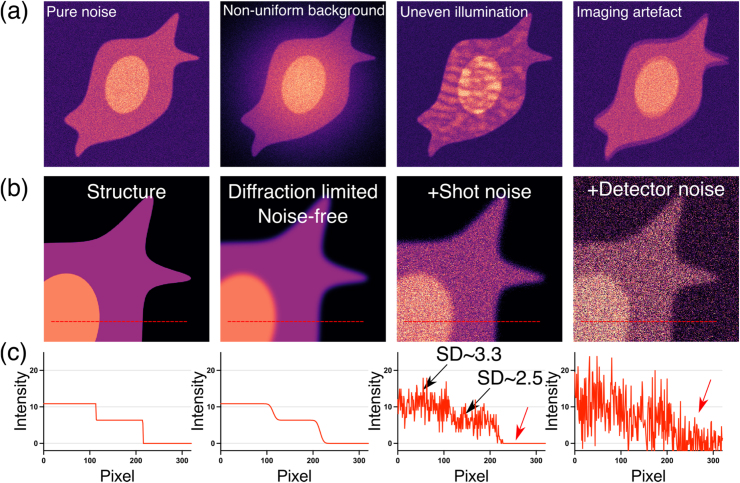
Noise and other image corruptions typically observed in fluorescence microscopy images. (a) Common image corruptions observed in fluorescence microscopy. From left to right: only noise, non-uniform background which may occur from e.g. vignetting, uneven illumination from e.g. laser illumination affected by speckle, imaging artefact such as the presence of a ghost image as shown here. (b) Starting from the true structure, the optics limit the resolution of the measurable image, leading to a smoothed diffraction-limited image. Upon measurement, the image is subject to signal-dependent Poisson noise (shot noise), and electronic detector noise. Only the right-most image can actually be experimentally measured. (c) Line profiles across the red dashed lines shown in (b), highlighting the loss of resolution (smoothed edges) and increasing levels of noise. Here, SD refers to the standard deviation of the shot noise, increasing with increasing levels of signal.

While the MSE is well suited for comparing e.g. the denoising performance of two different algorithms on a given image, it also comes with some caveats. The MSE can lead to surprising and unintuitive results when comparing the quality between two different images. Consider for example a low-exposure and a high-exposure recording of the same sample. While it would be reasonable to prefer the high-exposure image, the MSE metric will yield the opposite result, giving a lower value for the low exposure recording. The reason for this is that the reduced exposure leads to a reduced signal and in consequence to a reduced amount of shot noise (see Fig. 2c and Box 2), which depends on the intensity of the signal.

The solution to this paradox is to consider not the absolute amount of noise but the noise relative to the signal. The most commonly used metric that achieves this is the *peak signal to noise ratio (PSNR)*, which is defined as

$$\mathrm{PSNR}(x,s)=10\mathrm{lo}g_{10}(\frac{R{\left(s\right)}^{2}}{\mathrm{MSE}(x,s)}),$$

where R(s)=max(s)−min(s) is the range of values occurring in the clean image. A high PSNR corresponds to a close match between x and s (and therefore a low noise) and a low PSNR corresponds to a poor match.

At its core, the PSNR also measures the MSE, but it does so in relation to the range of values in the clean image. This metric will give the expected result and assign a lower value to the low-exposure image, which has a reduced amount of noise but also a reduced signal.

The PSNR is the *de-facto* standard used to quantify the amount of noise in an image and to judge the quality of denoised images. Note that while other metrics have been proposed, mainly to better match the human experience in judging image quality (e.g, structural similarity, SSIM (Wang et al., 2004)), we find that they seldom produce different results with respect to ranking the quality of denoising algorithms in fluorescence microscopy.

This review presents a basic description of noise in fluorescence microscopy and introduces the main DL strategies for denoising: *supervised* and *self-supervised* training. Although we will primarily focus on fluorescence microscopy as one of the most powerful live-cell microscopy techniques, the methods and the discussions around it also generally apply to other types of imaging methods such as phase contrast or electron microscopy, albeit with different noise characteristics. Finally, we give an overview of available DL-based denoising software and discuss important pitfalls.

## 2 Noise and the task of denoising

### 2.1 What is noise?

Every fluorescence microscopy image is an imperfect representation of the underlying structure that is being imaged. Multiple factors contribute to this imperfection: limited resolution (defined by the optics); uneven illumination or background; unwanted stray or out-of-focus-light reducing the image contrast; image artefacts; and, of course, noise (Fig. 2a). Here, we consider noise as the discrepancy between the true amount of light $s_{i}$ being measured at pixel i, and the corresponding measured pixel value $x_{i}$. We thus explicitly exclude other imperfections, as the ones mentioned above.

In fluorescence microscopy, the most dominant sources of noise are the *shot noise* (a fundamental type of noise due to the nature of light) and the *detector noise* (due to the electronics used to detect light, e.g., cameras or photodetectors, see Box 2 for more info) (Jezierska et al., 2016) (Fig. 2b). Viewed mathematically, the shot noise follows a Poisson distribution, which scales with the intensity of the signal. Somewhat paradoxically, this means that in absolute terms, bright pixels exhibit a larger noise level compared to darker ones (as seen in Fig. 2c, standard deviation comparison). However, relative to the pixel’s signal, the effect is more severe for low signal (Fig. 2c). Detector noise usually affects each pixel independently and uniformly, irrespective of the true underlying signal. It is often modelled as a simple additive Gaussian noise process. Box 2 presents a more mathematically oriented description of the noise model.

Box 2 **Noise model.**

The main noise components observed in microscopy are shot noise and the detector noise (see Fig. 2b). Given an underlying true signal $s_{i}$, representing the average number of photons at the detector in pixel i during an exposure, the measured experimental signal $x_{i}\;$(in Analog-to-Digital Counts, ADC) can be written as follows:

$$x_{i}=a\varphi (s_{i})+\varepsilon_{i}$$

Where $\varphi (s_{i})$ represents the shot noise-affected signal (which depends on $s_{i}$), a represents the conversion factor from the number of photons to ADC, as measured by the detector, and $\varepsilon_{i}$ represents the detector noise (in ADC). We will now take a closer look at both components:

**Shot noise** is due to the quantum nature of light, i.e., the fact that light can be understood as a stream of discrete photons. The shot noise-affected signal $\varphi (s_{i})$ corresponds to the number of photons measured by a pixel. Given an underlying signal $s_{i}$ at the pixel, the number of detected photons $\varphi (s_{i})$ will follow a Poisson distribution, centred around $s_{i}$ and is subject to random fluctuations with standard deviation $\sqrt{s_{i}}$. Therefore, as shown in Fig. 2c, and perhaps counter-intuitively, when the signal *increases*, the amount of shot noise *increases* as well, albeit less rapidly than the signal itself, leading to a better SNR overall.

**Detector noise**$\varepsilon_{i}$ is due to the electronic measurement occurring in the camera or photodetector. It often follows a Gaussian distribution with a constant standard deviation independent of the underlying signal. Gaussian noise can take negative values, so, to avoid negative values in our measured signal $x_{i}$, most cameras set a constant bias or offset value that is added to the signal. As a consequence, we can think of the detector noise as following a Gaussian distribution centred around this offset. The amount of noise will differ depending on the type of detectors used. Note that for some detectors and cameras, such as EMCCDs, this simple model of detector noise is inaccurate and we have to include an additional signal dependent component (the so-called excess noise).

Usually, both types of noise share a common property: they typically occur independently for each pixel, which means that the result in one pixel does not influence its neighbours. When we therefore think of the noisy recorded image x affected by shot noise and detector noise as being drawn from a probability distribution p(x|s), this **conditional pixel-independence** allows us to describe the distribution as product over pixels:

$$p(x|s)=\prod_{i=1}^{n}p(x_{i}|s_{i}),$$

where $p(x_{i}|s_{i})$is the distribution of a noisy value in pixel i given the clean underlying signal $s_{i}$. This feature of the noise is the key to self-supervised DL-based denoising.

Additionally, shot noise and detector noise are **centred around the true signal** $s_{i}$ (ignoring the background offset), so we will sometimes measure values $x_{i}$ that are higher and sometimes lower compared to the true signal $s_{i}$. However, if we were to average many acquisitions the result would converge towards the true signal. This is another way of saying that the expected value

$$E_{p(x_{i}|s_{i})}[x_{i}]=s_{i\;}$$

of the noisy observation is identical to the underlying signal. Noise2Noise (Lehtinen, 2018) training relies on this property to extract the denoised images from a pair of noisy images. By trying to solve the impossible task of using one noisy image to predict another, it effectively computes this expected value and solves the denoising task.

Self-supervised methods, such as Noise2Void (Krull et al., 2019), additionally have to rely on the conditional pixel-independence when they compute a similar expected value by trying to predict the value at a pixel from its surroundings.

Unfortunately, some cameras produce structured detector noise that violates this assumption. As a consequence, Noise2Void (Krull et al., 2019) can produce images that still contain a pattern of residual detector noise. In our experience, some sCMOS and, depending on the settings, EMCCD cameras are particularly susceptible in this regard, while laser scanning microscopes generally do not seem to have this problem at all. A solution for self-supervised learning with structured noise has been proposed in StructN2V (Broaddus et al., 2020).

The amount of noise in an image, given its true underlying signal level, can be quantified to compare denoising algorithm performance or simply assess the improvement observed in a specific dataset. Given a noisy image x and its corresponding clean counterpart s, a standard approach is to compute the *mean squared error* (MSE). Box 1 gives an in-depth description of the MSE as well as of another important noise metric, the PSNR, which is often used to compare denoising performance of different algorithms.

### 2.2 The task of denoising

Image denoising aims to provide a function f(x)≈s that takes a noisy image as input and returns an approximation of the true clean image as output. In order to successfully denoise an image, we need to rely on either of the two following components:

1. Our knowledge of the noise distribution in an image. We can use it to ask how likely a potential solution s would give rise to x (see Box 2). This rules out any solution that deviates too far from our observed image x.
2. Our knowledge of what clean images generally look like. We might e.g. expect an image to be smooth, and formally limit the possible denoised outcome to fall within a certain “smoothness” probability distribution called the *prior*.

Any denoising method is based on making assumptions about the noise and/or priors. While some classical methods explicitly use e.g. smoothness priors (Rudin et al., 1992, Haider et al., 2016), others do so only implicitly, e.g. by applying filters that tend to produce smooth results.

## 3 Denoising with deep learning

**Fig. 3:**
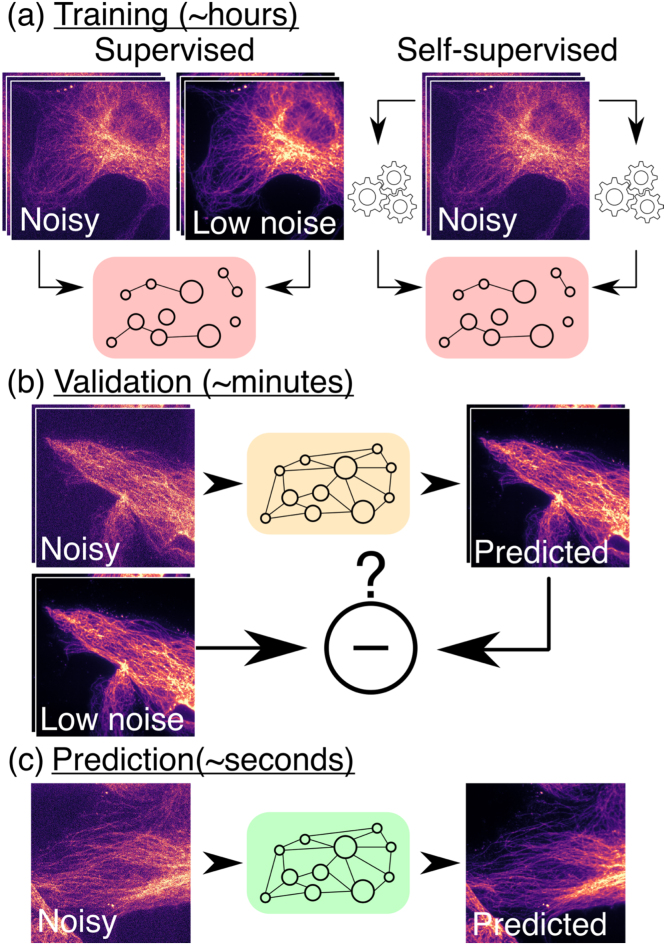
Main deep learning workflow for image denoising. (a) Supervised vs. self-supervised training schemes. (b) Validation of the model performance on a ground-truth dataset. (c) Once trained and validated, models can be used for predictions, often with excellent speed performance.

DL-based methods generally do not make explicit assumptions about the priors or noise models. Instead, they learn to expect specific patterns from their training dataset, which provide powerful *implicit priors*. They therefore learn what an image “should look like” for that specific dataset. For this reason, DL-methods are also referred to as *content-aware image restoration* as previously highlighted (Weigert et al., 2018), and therefore typically provide optimal results only for datasets similar to that used for training. To achieve this, a neural network trained for denoising directly implements the function f(x;θ)≈s, where θ generically represents the model parameters, called weights, which determine how the input image is transformed into the output image. Training a network corresponds to adjusting θ based on training data to optimise the network output quality Fig. 3.

The training strategies can be broadly classified into two categories, (i) *supervised* and (ii) *self-supervised*, and will determine what training dataset needs to be provided.

**Supervised training** requires a set of corresponding noisy input images and their clean counterparts. In this case, training consists of iteratively adjusting θ to minimise the discrepancy between the network output and the provided clean target image using for instance the MSE as a mathematical metric to be minimised (called the training loss). Supervised training works well in practice and is the default approach, leading to the highest quality results. However, the requirement for paired training data can be problematic. To achieve optimal results, it is essential that the training data is of the same type as the data to denoise. To avoid artefacts, the user should then ideally acquire new training data for each new experiment. One way to gather such training data is to record low-exposure and high-exposure images prior to starting a long experiment or using fixed samples. Supervised training is especially powerful as it does not rely on specific assumptions about the image degradation that needs to be corrected. It has therefore been extensively used in a wide range of cases, such as denoising of natural images or removing out-of-focus light (Weigert et al., 2018). For denoising, an innovative supervised training scheme was described by Noise2Noise (Lehtinen, 2018) where the network learns to predict one noisy image from another. The noise being in essence unpredictable, this approach results in denoising the input image.

On the other hand, **self-supervised training** allows training purely from single noisy images. The core idea is to use one part of the image as input and another as target. One example of this is the blind-spot approach (Krull et al., 2019, Batson and Royer, 2019), whereby, inspired by Noise2Noise (Lehtinen, 2018), the network learns to predict the intensity at a pixel i only from the surrounding pixels. Leveraging certain properties of the noise models distribution in microscopy images (see Box 2 for details), the network then learns to denoise the image. Today, self-supervised methods are capable of producing crisp results that can in many situations be comparable to supervised training (Krull et al., 2019, Goncharova et al., 2020), without the need for paired training dataset.

A downside of the self-supervised approach is that the assumption of pixel-independent detector noise may in some cases not hold, depending on the detector used. Such structured imaging noise (e.g., streaks or patterned noise) is not removed and can become clearly visible in the network output.

## 4 Denoising tools using deep learning

A number of DL-based denoising tools are now available to biomedical researchers without the need for expert programming skills. For instance, Noise2Void (Krull et al., 2019) can be directly trained and used for predictions using the popular image analysis software Fiji/ImageJ (Abramoff et al., 2006, Schindelin et al., 2012). ZeroCostDL4Mic (Chamier et al., 2021) is also becoming popular among biomedical researchers interested in testing and exploring the use of DL for their microscopy studies. The ImJoy (Ouyang et al., 2019) platform provides an easy-to-use user interface for a range of neural networks including CARE (Weigert et al., 2018). In addition, commercial tools from microscope manufacturers are becoming available, e.g, Nikon’s Denoise.AI and Zeiss’ Apeer, or from DL-focused image analysis platforms such as Leica’s Aivia.

Additionally, denoising is also commonly used in the field of natural images such as photography or astronomy and a range of DL-based tools are also available for these, such as Topaz Labs Denoise AI. We however do not recommend using these tools for microscopy images since they are developed specifically using photographs as training datasets, therefore containing significantly different noise and data structure from what is in microscopy images.

Table 1 presents an overview of the tools for DL-based denoising which are currently available within user-oriented platforms. We highlight the type of instructions that are available for the user to exploit the methods, as well as the type of software, as was previously done for segmentation software (Lucas et al., 2021). Beyond simply denoising 2D images, many of the implementations described here have the capabilities of working on 3D dataset or even concomitant denoising of multiple channels, (see Table 1 for details). DeepCAD, a recent denoising implementation based on 3D U-Net (Çiçek et al., 2016), efficiently improves the SNR of time-course calcium imaging (Li et al., 2021). Here, using additional information from the context of the pixels in any relevant dimensions (3D, time or other channels for instance) to denoise often greatly improves denoising performance but at the expense of longer training times and the need for larger training datasets.

**Table 1:** Overview of the currently available user-oriented tools for DL-based denoising. Open-source and commercial tools are available. T: training, P: prediction/pretrained model. *DenoiSeg can provide concomitant denoising and image segmentation, but only requires that some of the data be manually segmented for learning both tasks.

Method	Training type	Capabilities	Integration	Instructions	Comments	Software type	Link	Reference
CARE	Supervised	2D, 3D, multi-channel	Fiji (P), ZeroCostDL4Mic (T&P), ImJoy (T&P)	Website, video tutorials, GitHub page	Can perform a range of image restoration tasks	Free, open-source	https://csbdeep.bioimagecomputing.com/tools/care/	Weigert et al., (Weigert et al., 2018)
Noise2Void	Self-supervised	2D, 3D, multi-channel	Fiji (T&P), ZeroCostDL4Mic (T&P), Apeer (P), ImJoy (T&P)	Website, video tutorials, GitHub page	Can be trained directly on the images to denoise	Free, open-source	https://csbdeep.bioimagecomputing.com/tools/n2v/	Krull et al., (Krull et al., 2019)
DecoNoising	Self-supervised	2D	ZeroCostDL4Mic (T&P)	Website, video tutorials, GitHub page	Can be trained directly on the images to denoise, performs deconvolution simultaneously	Free, open-source	https://github.com/juglab/DecoNoising	Goncharova et al., (Goncharova et al., 2020)
3D-RCAN	Supervised	2D, 3D	Aivia (T&P), ZeroCostDL4Mic (T&P)	Website	Extension of RCAN network, can do resolution improvement	Commercial, code open-source	https://www.biorxiv.org/content/10.1101/2020.08.27.270439v1	Chen et al., (Chen et al., 2020)
Noise2Noise	Self-supervised	2D	Apeer (T&P)	Website	Requires pairs of noisy images	Commercial	https://www.apeer.com/app/modules/AI-Image-Denoising/d551013b-258a-40b0–84aa-f710c6cf02ca	Lehtinen et al., (Lehtinen, 2018)
DenoiSeg	Partially supervised*	2D	Fiji (T&P), ZeroCostDL4Mic (T&P)	Website, video tutorials, GitHub page	Provides denoising and segmentation	Free, open-source	https://csbdeep.bioimagecomputing.com/tools/denoiseg/	Buchholz et al., (Buchholz et al., 2020)
Denoise.AI	Supervised	2D	NIS Elements (T&P)	Website	Unknown architecture	Commercial	https://www.microscope.healthcare.nikon.com/products/confocal-microscopes/a1hd25-a1rhd25/nis-elements-ai	unknown

Beyond the task of denoising, a number of neural networks were developed to provide additional functions. For instance, CARE and 3D-RCAN (an extension of the RCAN architecture (Zhang, 2018)) are actually general image restoration networks that can improve resolution, noise levels or even remove image artefacts. DenoiSeg (Buchholz et al., 2020) performs both image denoising and segmentation simultaneously, leveraging an improved segmentation performance from denoising the image. It also has the advantage of not requiring all the training data to be segmented therefore minimising the effort in curating the training data. DecoNoising (Goncharova et al., 2020) performs both image denoising and deconvolution from 2D data as is often done to remove out-of-focus signal and improve image contrast. The user only needs to provide a representative point-spread function (PSF) of the imaging system that can either be measured or simulated for the deconvolution.

Beyond the methods shown in Table 1, other methods have been demonstrated but have not yet been integrated within the user-oriented platforms that we highlight here. For instance, Noise2Void has been evolved to take into account specific noise distributions to improve performance (PN2V (Krull et al., 2020)) or to cope with structured noise (StructN2V (Broaddus et al., 2020)).

Also, DivNoising (Prakash et al., 2020) provides not just a single image output but a whole distribution of probable images, therefore highlighting regions of the image that are predicted with lower certainty.

Commercial platforms such as those shown in Table 1 often use approaches described in published scientific papers, but in other cases, such as Nikon’s Denoise.AI, little information about the network architecture or the training dataset used are available.

## 5 Pitfalls and limitations

Although adequately trained DL-based methods have been shown to outperform classical approaches and tend to produce results closer to the true signal when assessed with quality metrics such as PSNR and SSIM (Weigert et al., 2018, Krull et al., 2019), the application of DL will inevitably introduce artefacts and distort pixel intensities, potentially in non-linear ways, which may render subsequent intensity-based quantification prone to errors. Eventually, the performance of any denoising approach should be assessed based on the final goal of the analysis pipeline. For instance, when performing ratiometric analysis of two channels, treating both channels independently for denoising will almost certainly void the possibility for any quantification from calculating the ratio of the denoised images. In this case, it is likely that the quantification may be better performed before denoising. Therefore, we do not recommend, at this stage, performing intensity-based quantification on denoised images but rather to go back to the raw as much as possible to avoid artefacts. This observation is true for any sophisticated denoising approach, and not just DL-based. There is however scope for performing simultaneous denoising of multiple channels using DL which would potentially better retain relative intensities. The effect of denoising on the quantifiability of the pixel intensities is a matter of ongoing research. However, the denoised images can be used to obtain certain features of the image, such as a more robust segmentation or localisation of objects that is usually the basis for subsequent intensity measurements.

**Fig. 4:**
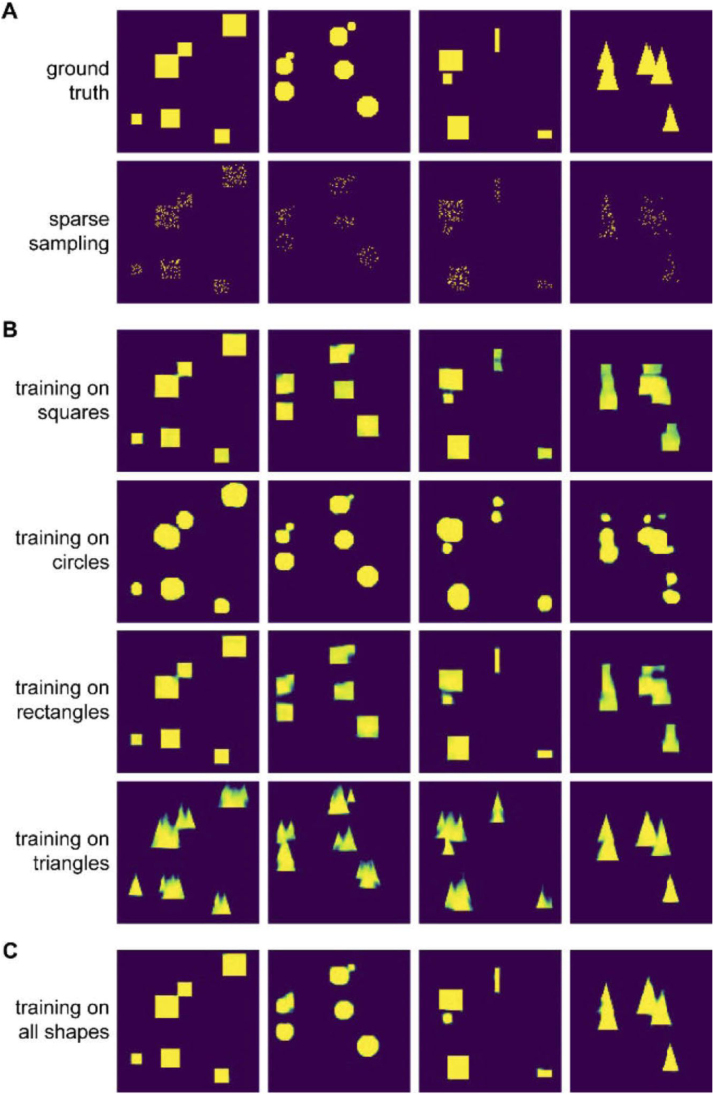
Deep learning and *structural priors*. Artefacts due to structural priors can appear when using data that are significantly different from those used at the training stage. The network is likely to produce the patterns it saw during training, even when they are not present in the data.

Here, it is essential to remember that the quality of DL-based denoising entirely depends on the quality of the training data. Based on the training data, the network learns which patterns it can expect to find in an image. A network trained on data significantly different from the data it is applied to is likely to hallucinate structures not present in the sample. Fig. 4 highlights this phenomenon. Here, the model clearly learns to expect specific shapes, (implicit *structural prior*).

Figure reprinted with permission from ​Möckl et al. (2020) © The Optical Society.

Ideally, training data need to be acquired in the exact same conditions as the data to process downstream of training and validation. Self-supervised methods have an advantage in this respect, as they allow training on the very same data that is to be denoised.

## 6 Conclusion

Recent DL applications to bioimage analysis demonstrate that DL-based denoising is pervading the bioimaging community and is proving useful for a range of tasks. Here, we provide an overview of DL-based denoising as a group of methods that is particularly powerful to live-cell imaging. We have considered the advantages and disadvantages of supervised and self-supervised denoising methods and presented the currently available networks, with some of them providing additional functions beyond denoising (e.g., segmentation, deconvolution, distribution of probable output image). Importantly, self-supervised approaches can allow denoising from as little as a single noisy image without the need for providing ground truth at the training stage.

However, as with any computational approach, denoising should be applied with care and results checked for potential for artefacts with validation steps. While the performance of DL-based methods can outperform classical approaches when comparing overall image quality metrics (Box 1), the artefacts originating from DL-based denoising, notably from the embedding of structural priors (Fig. 4), are an active topic of research, and we can hope that community efforts will lead to their better understanding in the near future. In particular, there is a need for agreed upon quality metrics (Chen et al., 2021) and standard dataset for performance testing and comparison (Qiao et al., 2021) allowing us to assess any new methods.

We hope the future will bring more powerful denoising methods, especially benefitting from partially supervised approaches and further mitigating the heavy burden of curating training datasets. The application of transfer learning (Wang et al., 2021) will notably allow minimising the occurrence of artefacts and allow the use of smaller training datasets for building models that will be optimised for specific dataset. It will be interesting to further study how networks for denoising large 3D multi-colour simultaneously, as is already partially done with CARE (Weigert et al., 2018), can utilise the co-dependence between different fluorescence channels, within volumes, or even using behaviour in time. Currently, a range of tools have the capability to handle 3D dataset (see Table 1) which immediately gives the possibility to denoise 2D time-course data (2D+t) by replacing the third dimension by the temporal dimension. Despite 3D+t (4D) or multi-channel 3D+t (5D) data potentially providing better context for denoising, they have not been exploited so far, potentially due to the very large data dataset necessary for this and their corresponding computational requirements at training stage.

Importantly, the noise model and methods for denoising described here have ignored the way in which such images were obtained at the microscope. In fact, wide-field, confocal or 2-photon microscopy images will generally be well described by the above noise model, but specific detectors or acquisition conditions may lead to the presence of structured noise in the data (e.g. stripes due to non-homogenous detector properties across pixels of CMOS cameras), which will become more challenging to denoise especially for self-supervised approaches (see Box 2 for further details).

Another interesting perspective is to move beyond convolutional neural networks (CNNs) and use generative DL models, such as Variational Auto-Encoders (VAE) (Prakash et al., 2020, Kingma and Welling, 2013) or Generative Adversarial Networks (GANs) (Goodfellow, 2014). These types of models can in principle be trained purely from noisy data and will allow us to embed our knowledge of noise and image formation within a microscope.

Finally, although DL-based denoising can be very powerful, it is not always the most efficient way to achieve a particular image analysis goal. In fact, it all depends on what is required from the data *subsequently* to the denoising step. If simple denoising such as Gaussian filtering does not compromise downstream analysis or quantification, it may not make much sense to seek any sophisticated denoising approaches, whether DL-based or not. The performance of an algorithm and when a denoising approach is good enough should always be assessed with respect to the end point of the analysis pipeline.

In our experience, DL-based denoising should however always be at least considered as an option thanks to its potentially high data specificity and performance, but there may in fact be cases where DL-based denoising should be avoided. Such cases may include scenarios when accurate intensity-based quantifications are performed or when it is very challenging to generate training datasets that are representative enough of the data subsequently analysed. The latter case may silently lead to artefacts that are typically difficult to detect.

## Declaration of Competing Interest

The authors declare that they have no known competing financial interests or personal relationships that could have appeared to influence the work reported in this paper.
